# Free-breathing myocardial T_1_ mapping using magnetization-prepared slice interleaved spoiled gradient echo imaging

**DOI:** 10.1186/1532-429X-17-S1-W7

**Published:** 2015-02-03

**Authors:** Sébastien Roujol, Jihye Jang, Tamer A Basha, Sebastian Weingartner, Sophie Berg, Reza Nezafat

**Affiliations:** 1 Department of Medicine (Cardiovascular Division), BIDMC / Harvard Medical School, Boston, MA, USA; 2 Computer Aided Medical Procedures, Technische Universität München, Munich, Germany; 3 Computer Assisted Clinical Medicine, University Medical Center Mannheim, Heidelberg University, Mannheim, Germany

## Background

Quantitative myocardial T_1_ mapping and extracellular volume fraction (ECV) show promise for non-invasive assessment of cardiomyopathies. Most available T_1_ mapping sequences use a single slice breath-hold acquisition with balanced steady state free precession (b-SSFP) readout [1]. However, b-SSFP readout is sensitive to B_0_ field inhomogeneity and is potentially T_2_ dependent [1]. In this study, we sought to investigate the feasibility of a free breathing multi-slice T_1_ mapping sequence using slice-interleaved spoiled gradient echo (GRE) imaging.

## Methods

The proposed sequence used multiple inversion recovery (IR) experiments. In each IR experiment, a non-selective inversion pulse is applied and followed by the acquisition of 5 slices over the next 5 heart beats, and 3 rest cycles [2]. This IR experiment is repeated 5 times using different slice orders to obtain signal samples at TI, TI + 1 RR, TI + 2 RR, TI + 3 RR, TI + 4 RR. This block of 5 IR experiments is finally repeated using a different TI value. The fully recovered longitudinal magnetization is also initially acquired for each slice without any IR pulse (∞ image). Respiratory motion was corrected using prospective slice tracking and retrospective image registration. ECG-triggered single shot acquisitions were used with GRE readout (TR/TE/α=4.3/2.1ms/10˚, FOV=280×272 mm^2^, voxel size=2×2 mm^2^, slice thickness=8 mm, 5 slices, 43 phase-encoding lines, linear ordering, 10 linear ramp-up pulses, SENSE factor=2.5, half Fourier=0.75, bandwidth=382Hz/pixel). For comparison, MOLLI [3] was acquired with a b-SSFP readout and similar parameters (except TR/TE/α=2.6/1.3ms/70°, 1 slice, bandwidth=1785 Hz/pixel). Imaging was performed on a 1.5 T Philips scanner. T_1_ accuracy, precision, and reproducibility were evaluated in simulations and phantom. In-vivo spatial variability and reproducibility of native T_1_ mapping was measured in 11 healthy adult subjects (35±21y, 4 m), imaged 5 times with each sequence. Three of these subjects were also imaged at ~15min after contrast injection to demonstrate the feasibility of ECV mapping.

## Results

The proposed sequence provided improved accuracy and similar precision than MOLLI in both simulation and phantom experiments (accuracy: p=0.01; precision: p=0.16). MOLLI was more reproducible in phantom (p<0.001). In-vivo, the proposed sequence yielded higher native T_1_ times than MOLLI (1094±24ms vs. 1010±27ms, p<0.001) with similar spatial variability (58±7ms vs. 61±9ms, p=0.44) and reproducibility (25±9ms vs. 17±8ms, p=0.15). ECV measurements were 0.21±0.01 using the proposed sequence.

## Conclusions

Free breathing multi-slice T_1_ mapping using a magnetization-prepared slice interleaved spoiled GRE imaging is feasible and yields similar in-vivo precision/reproducibility as MOLLI but with improved accuracy. In addition, the proposed sequence allows simultaneous imaging of 5 slices within free-breathing in 100 sec.

**Figure 1:**
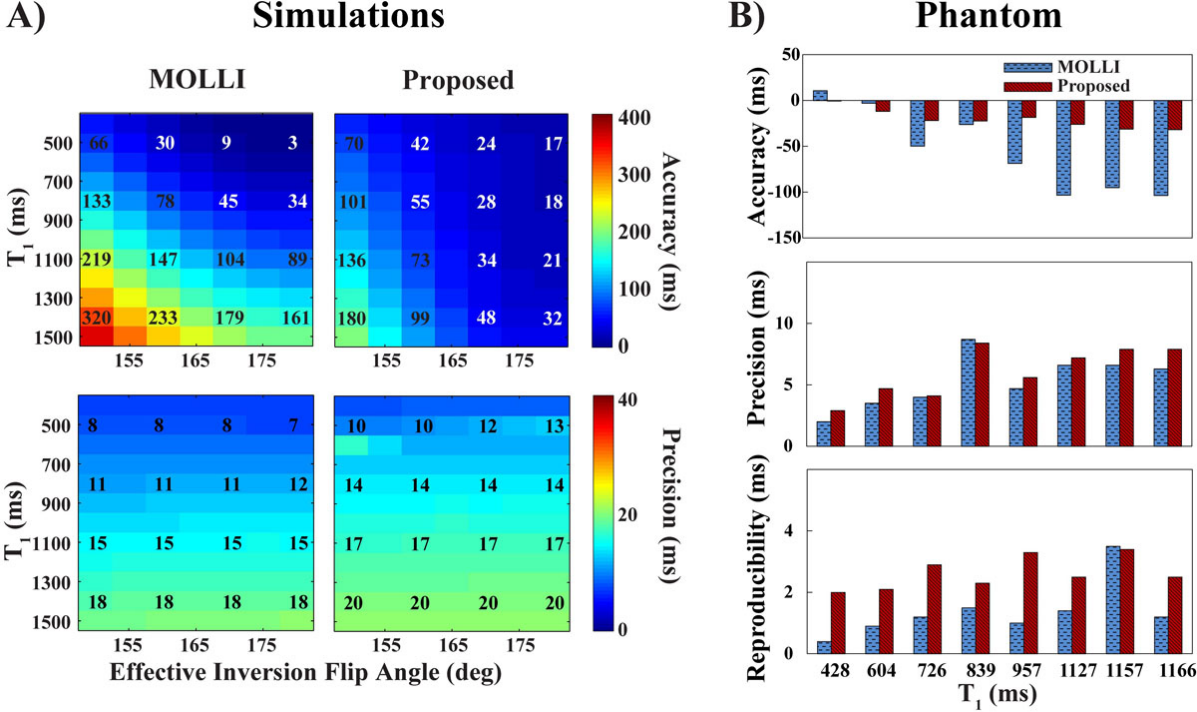
Accuracy, precision and reproducibility of the proposed sequence obtained in Monte Carlo simulation (20,000 repetitions, fixed T_2_ of 50 ms, SNR corresponding to 50 in the ∞ image) (a) and phantom experiments (set of vials with NiCl2 doped agarose, 15 repetitions of the sequence) (b). Results were compared to the MOLLI (5-(3)-3 scheme) sequence. Accuracy was measured in each vial as the difference between spin echo T_1_ measurements and the average T_1_ over all 15 repetitions. Precision was measured in each vial as the average (over all 15 repetitions) of the standard deviation of T_1_ within a vial. Reproducibility was measured in each vial as the standard deviation (over all 15 repetitions) of the mean T_1_ within a vial. Improved accuracy and similar precision were achieved using the proposed sequence in both simulations and phantom experiments. T_1_ mapping reproducibility was slightly decreased with the proposed sequence.

**Figure 2:**
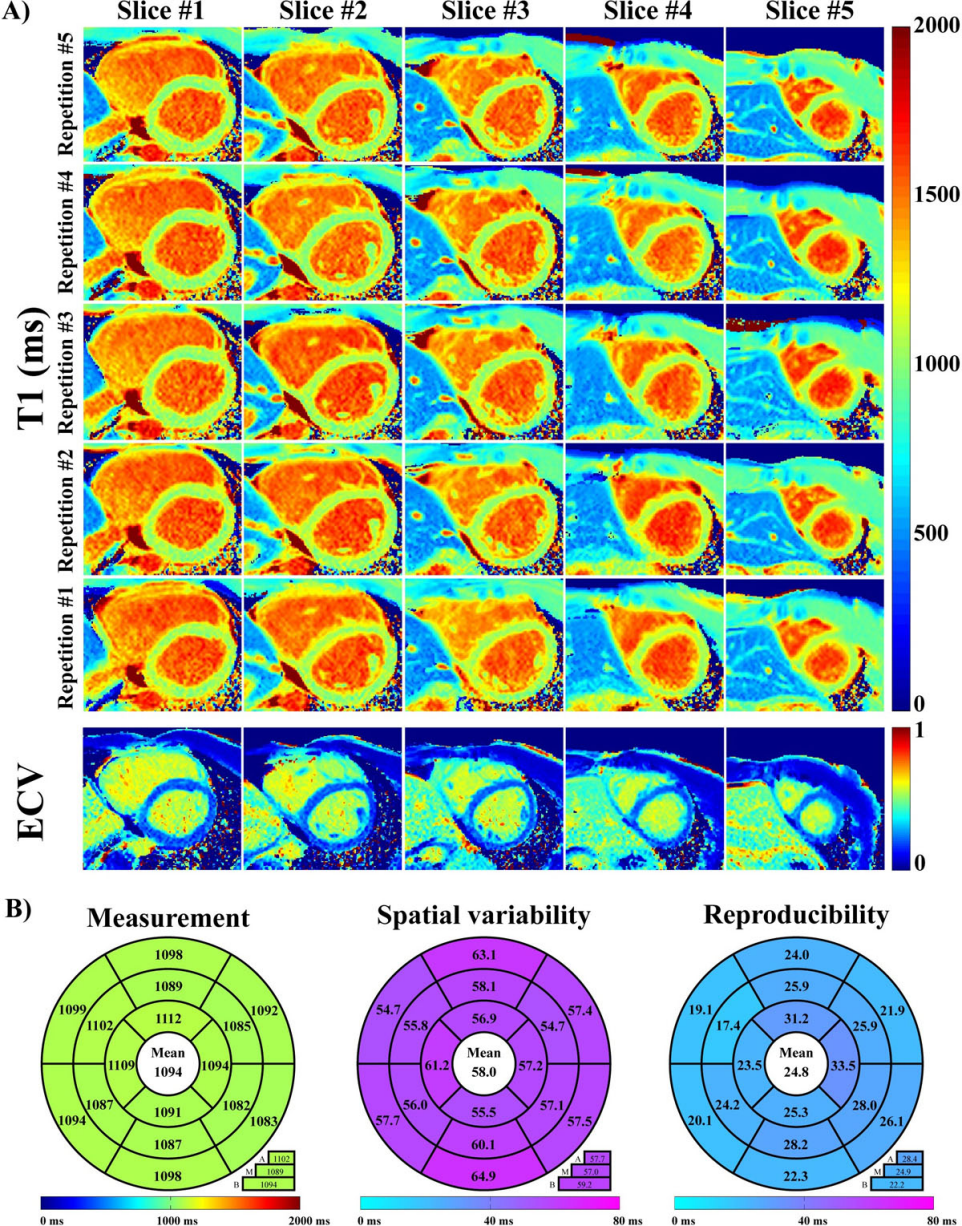
In-vivo native T_1_ and ECV mapping using the proposed sequence. Example of multi-slice T_1_ maps and ECV maps obtained in one healthy subject is shown in (a). Homogeneous T_1_ map quality was achieved in all slices for all five repetitions. Homogeneous ECV map quality was also observed through all slices. Native T_1_ measurements, spatial variability, and reproducibility obtained using the proposed sequence, are reported in average over all subjects in (b). Each metric was quantified using a 16 myocardial segment model in all subjects by analysis of the three mid-ventricular slices. Spatial variability was measured for each segment as the average (over the five repetitions of all subjects) of the standard deviation of T_1_ measurements within that segment. Reproducibility was measured for each segment as the average (over all subjects) of the standard deviation (over the 5 repetitions) of the mean T_1_ time of that segment.
